# Factors contributing to antiretroviral drug adherence among adults living with HIV or AIDS in a Kenyan rural community

**DOI:** 10.4102/phcfm.v9i1.1343

**Published:** 2017-07-31

**Authors:** Mary T. Kioko, Anne M. Pertet

**Affiliations:** 1 Department of Nursing, Kenya Medical Training Institute, Kenya; 2 Department of Community Health, Great Lakes University, Kenya

## Abstract

**Background:**

Antiretroviral (ARV) adherence of ≥ 95% is recommended for suppressing HIV. However, studies have shown that the ≥ 95% recommended level is rarely achieved.

**Objective:**

This cross-sectional community-based study sought to assess factors contributing to ARV drug adherence among adults living with HIV or AIDS.

**Setting:**

The study was conducted in a rural community in Machakos County, Kenya.

**Methods:**

The questions used for the study were adapted from the Patient Medicine Adherence Questionnaire (PMAQ), a tool grounded in the Health Belief Model. Adherence to ARV was measured using self-reports and pill counts. The perception social support was measured with a 5-point Likert scale, whereas the type and the number of side effects experienced were recorded using ‘yes’ and ‘no’ questions. We used the chi-square test to test associations and binary logistic regression to assess factors explaining dose adherence to ARV.

**Results:**

The levels of adherence of 86% using self-reports were significantly higher (*p* < 0.001) than the pill count of 58.6%. The immediate family was rated high in providing social support (3.7 ± 0.6) followed by social support groups (3.1 ± 0.8). A binary logistic regression analysis was conducted to predict ARV adherence (adherent, non-adherent) using social support, side effects and marital status as explanatory variables. The Wald criterion demonstrated that marital status (*p* = 0.019) and burden of side effects (*p* ≤ 0.001) made a significant contribution to the prediction of ARV adherence.

**Conclusion:**

The burden of side effects and being a divorcee are primary predictors of ARV adherence.

## Background

High levels of adherence (≥ 95%) are strongly correlated with disease suppression and reduction of morbidity and mortality rates among people infected with HIV and can suppress HIV to undetectable levels.^1,2^ However, such high levels of adherence have been difficult to achieve among persons living with HIV or AIDS (PLWHA), even in developed countries like United States where the use of antiretroviral therapy (ART) among HIV-infected adults was 85%.^3^ Research, however, shows that there is little consensus on the minimum threshold of adherence needed for virologic suppression.^4,5^ Studies using self-reports where a patient voluntarily reports to the health care provider the doses of antiretrovirals (ARVs) missed over a period of time have reported varied adherence levels. A study from eastern Ethiopia recorded adherence levels of 85%.^6^ However, other African studies have reported lower adherence levels. These include 70% in Botswana, Tanzania and Uganda^7^; 62.6% in Togo^8^; 68% in Kenya^9^; and 25% in South East Nigeria.^10^ Studies that have used pill counts came up with much lower levels. These include 67% using pill count at a hospital set-up^11^ and between 28.3% and 69.8% using pill count and pharmacy refill methods in the United States.^12^ These statistics show that the ideal adherence levels (≥ 95%) were not achieved, and self-reports tended to give higher prevalence levels. Various factors have been associated with non-adherence. These include patient-related factors, inconvenient dosing frequency, dietary restrictions, pill burden, side effects, social support, patient–health care provider relationships and the system care. This study explores factors contributing to ARV adherence in a rural Kenyan community.

Evidence on the relationship between sex and adherence has been inconclusive. In a field-based study conducted in Zambia, adherence was associated with being female or having a spouse on ARVs (adjusted odds ratio [AOR] 3.3 and 5.0, respectively). These studies showed women were three times more likely to adhere to drugs than men.^13^ A study from Nigeria showed that being single was associated with non-adherence.^10^ Some studies in Kenya found no significant association between demographic factors (age and gender) and adherence,^9,14^ although a Nigerian study found that younger age groups were more adherent to ARV than older age groups.^13^ The lower level of general education and poorer literacy have also been shown to impact negatively on patient’s ability to adhere in the same studies.^14,15^

Social support is the help that a person receives from people in his or her social networks.^7^ Family members and friends can provide support to people living with HIV or AIDS in different ways which enable them to adhere to their ARV treatment. Family members have been shown to give support by reminding the patients to take their drugs and to get refills and accompanying them to appointments.^16,17,18^ Studies done in Thailand have shown that family communication was a significant predictor of adherence.^19^ Family members have been found to be the key source of material support (food, clothing and finance) to people living with HIV or AIDS.^17,20^ Studies conducted in Uganda, Tanzania and Botswana found no difference in adherence between those who had social support and those who did not have.^7^ Other researchers have found social support was not a significant predictor of adherence.^19^ Patients who reported that they were able to remember the routine schedule for taking their medicines with the help of significant others had higher adherence levels of ≥ 95% than those who did not have this support.^16^ Qualitative studies show that patients tend to associate social support and adherence.^20,21^

Antiretroviral drugs have been found to produce both minor and adverse side effects. Qualitative studies have shown that side effects have consistently been associated with decreased adherence.^16,21,22^ Few quantitative studies have been conducted on relationships between side effects and adherence. Results from a quantitative study conducted in South Africa showed no significant relationships between adherence and the intensity of symptoms. Logistic regression analysis indicated that the greater number of symptoms were not associated with lower adherence, which implies that despite the side effects patient were able to continue being adherent.^23^

Evidence from these studies shows that relationships between social demographic factors, social support and side effects with adherence have been inconclusive. The majority of the studies were qualitative, descriptive, hospital- or facility-based and used self-reports to measure adherence. There are virtually no studies using the pill count at the household level and few analytical studies on relationships between social support and side effects with ARV adherence. In our study area, data from the community care centre (CCC) show low adherence levels despite the intense HIV or AIDS interventions. The education does not particularly emphasise social demographics, social support and side effects. Better knowledge of these factors may inform the development of localised, practical and achievable interventions which will improve adherence.

Therefore, the aim of this study was to determine factors contributing to ARV adherence in order to come up with information that can be used to develop interventions to improve ARV drug adherence.

## Methodology

### Study area, study population and sampling

This study was carried out in Machakos County which has a population of 1 155 957 people (49% male population and 51% female population). The overall prevalence of HIV or AIDS is 5% with the prevalence among women being higher (6.8%) than that of men (2.9%). About 73% of people had never tested for HIV. About 25% of the adults are enrolled for care.^24^ The majority of HIV or AIDS patients use Machakos level five comprehensive care clinic for treatment refill, care and testing. Some supportive care is provided through six community units and support groups. The International Care and Treatment for AIDS Programme (ICAP) also provides HIV or AIDS care and ARVs. This study was conducted in Central Division, Machakos County, Kenya, where Machakos level five is located. The division also has several other health facilities. The study population comprised 417 male and 833 female adult PLWHAs registered in the CCC who were confirmed HIV positive through an HIV test and had been on ARV drugs for at least 6 months before the date of data collection. The study excluded seriously ill patients. Fischer et al.’s formula (*Z*^*2*^*pq/d*^*2*^)^25^ was used to calculate the sample size. We used a risk and degree of variability (*p*) of 0.64, level of confidence (*Z*) of 1.96 and level of precision (*d*) of 0.05. Because the study population was < 10 000, the sample was adjusted, and a non-response rate of 5% was added to arrive at a minimum sample size of 307. The stratified random sampling method was used to sample 102 men and 205 women from the list of 417 men and 833 women registered at the CCC. The community health workers (CHWs) were used to assist in identifying the household where the sampled respondents resided. Collection of information at the household represented real-life settings thus increasing the generalisability of the results for larger study population.

### Study design and data collection

A cross-sectional study with an analytical design was adopted for this study to demonstrate relationships. The Health Belief Model, which states that both internal and external (significant endorsement of others) motivation are necessary to produce change, was used to guide this study.^26^ The study subjects included all adults living within the study area who were confirmed to be HIV positive through an HIV test and had been on ARV drugs for at least 6 months prior to the date of data collection. ARV adherence was measured using both self-report or patient recall and pill count in the household. For self-reports, the respondents were asked whether they had missed taking the drug in the past 7 days and the number of drugs missed was recorded. Pill count method involved checking the number and date the pills were prescribed from the container and counting the doses of medication in the pill container. The quantity of ARV drugs prescribed minus pills counted and duration in days was used to calculate doses taken. The level of adherence was calculated using the number of doses reported to have been taken divided by total doses which were supposed to be taken within that duration and then multiplied by 100. These were then categorised as ≥ 95% or < 95%. Social demographic characteristics were limited to age, sex, the level of education and marital status. A 5-point Likert scale was used to measure perception of the support received from the various sources – immediate family, extended family, friends, support group, community source – and type of support – food, material, financial support and information on ARVs. The scale included strongly agree = 5, agree = 4, disagree = 3, strongly disagree = 2 and no opinion = 1. Data on side effects were measured by asking the respondents if they had experienced side effects and the type of the side effects experienced within the previous 7 days by yes and no questions. For this study, medicine-related side effects were categorised according to patient or ARV user and biomedical perspectives. Data were collected using interviewer-administered structured pre-coded questionnaire with closed questions adapted from the Patient Medicine Adherence Questionnaire (PMAQ), a tool that is grounded in the Health Belief Model.^27^ The tool covered perceived barriers about a patient’s social support network, knowledge on ARV drugs, attitudes and perceived qualities of the ARV medicines, a patient’s schedule and memory. However, for this study, the questionnaire only had four sections: social demographic factors, knowledge of the name of the ARV drug(s) being taken, social support and ARV drug side effects. Research assistants were high school graduates who spoke both English and Kamba (the local oral dialect) fluently. They were recruited and trained to administer questionnaires at the household level. To ensure the reliability of the data, the data collection instrument was pretested on 10% of study participants who were not included in the study. The pretesting sought to establish time taken to administer the questionnaire and checked for ambivalent or sensitive questions, the right use of language, and the relevance of questions. The changes and recommendations from the pretesting exercise were incorporated into the final tools. Informed consent and oral permission were obtained from each participant before filling in the questionnaire. The respondents were assured of the confidentiality of their information. Respondents’ names were kept anonymous. Information collected from participants was kept in a password-protected network file in the computer which was accessible only to the principal researcher.

### Data analysis

Excel was used to enter and clean the data. Data were analysed by SPSS (version 19) statistical software program. The dependent variable was ARV adherence, whereas the independent variables were age, sex, marital status, education, (social demographic variables), drug side effects and social support. The number of side effects was added up for each to calculate the ‘burden of side effects’ or concentration of side effects. A composite variable of social support was constructed from the Likert scale; the number of side effects, age and adherence were treated as continuous variables to test for correlations. The continuous variables were categorised into dichotomous variables to test for relationships using chi-square and binary logistic regression analysis. Proportions were used to estimate the adherence rate, the percentage of the patient by demographic factors and the types of side effect experienced. The 50th percentile was used to categorise the continuous data into two categories. Chi-square tests were used to test the association between variables that were categorised and adherence levels (≥ 95 and < 95). A *p* < 0.05 was considered as significant. Descriptive statistics (means and standard deviation) were used to compare mean values of continuous variables among subclasses of sources and types of social support. The ANOVA test was used to test whether the difference between the means of these sub-class was significant. The analysis was conducted to demonstrate the correlation between age, social support, side effects and adherence rate using Pearson’s correlation for continuous data which was normally distributed and Spearman’s rank for data which was skewed or ordinal. The correlation coefficient (*r*) was used to describe the strength of the supposed linear association between the continuous variables. To investigate the relative importance of the independent variables which showed some association about the dependent variable, and any confounding between them, the variables were fitted together in a binary logistic regression model.

### Ethical consideration

Authorisation to conduct the study was obtained from Great Lakes University of Kisumu Institutional Ethical Review Committee (GREC/152/21/2014) and Machakos County Medical Officer of Health.

## Results

### Social demographic characteristics of the study participants

The results on social demographic characteristics are shown in Table 1. A total of 301 patients participated in the study. Their mean age was 40.4 ± 10.8 years ranging from 22 to 64 years. The majority of the participants were women (62.8%). Most (60.1%) either had no education or had only 8 years of basic education. Only 7.3% had tertiary education. About one-half (58.1%) of the study participants were married.

**TABLE 1:** Distribution of social demographic variables of people living with HIV or AIDS in Machakos County, Kenya, 2015, *N* = 301.

Characteristics	Variables	Percentage
Sex	Male	37.2
	Female	62.7
Education	None	8.3
	Basic	51.8
	High school	32.6
	Tertiary	7.3
Marital status	Married	58.1
	Single	21.9
	Divorced	7
	Widowed	13
Age	20–29	5
	30–39	50
	40–49	30
	50–59	10
	60 and above	14

### Knowledge of the name of antiretroviral drug used

Information from the pill container label showed that 35.8% of the participants were on a combined therapy of AZT + 3TC + NVP, 34.7% were on TDP + 3TC + NVP, 20.5% were on ABC + 3TC + EFV and 9% were on TDF + 3TC + alluvia. When asked whether they knew the name of the drug they were using and how it was supposed to be taken, 63% of the respondents did not know the name of the drug they were using. All the respondents knew the consequences of not taking the drugs as instructed. The consequences reported included drug resistance 63%, treatment failure 12% and death 25%. There was a significant relationship between education levels and knowledge of the name of the ARV drugs (*χ*^2^ = 13.067, df = 3, *p* = 0.004). Only 3% of the patients with no education category knew the name of the drug they were using. There was also a significant relationship between sex and knowledge of the ARV drugs (*χ*^2^ = 4.0, df = 1, *p* = 0.045). Women tended to be more knowledgeable of the name of drugs. Marital status and age were not related to knowledge of the drug (*p* ≥ 0.05). There was no significant association between the knowledge of drug and adherence (*p* = 0.58).

### Antiretroviral adherence

**FIGURE 1:**
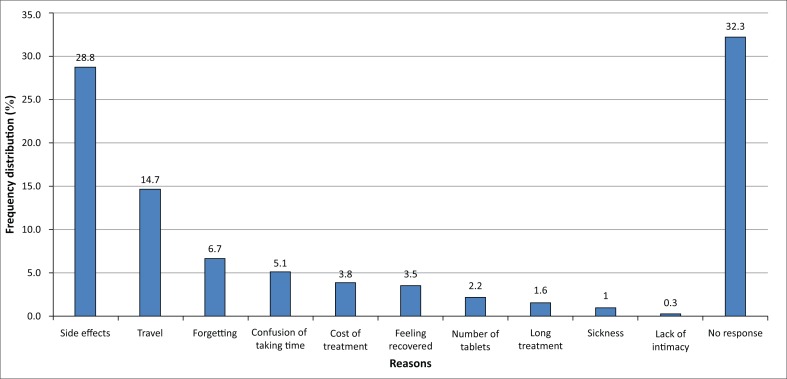
Frequency distribution (%) of reasons for missing antiretroviral drugs reported by people living with HIV or AIDS in Machakos County, Kenya, 2015, *N* = 301.

Results indicate that ARV adherence level measured using self-report was 27% higher than using the pill count. The adherence level using self-report was 85.6%. Of those who had missed the drug (14.4%), 67.7% had missed only 1 drug, whereas the rest had missed > 1 drug. Only 43.5% of the respondents were taking prescribed pills as instructed, whereas the majority (56.5%) were taking more or fewer pills per intake. Using pill count, only 58.6% of the respondents had reached the recommended level of ARV adherence of ≥ 95%. The majority (82.5%) used short message service (SMS) to remind them to take their drugs; 1% used watches; 6% were reminded by a family member, whereas 10.5% moved with their dose. About a third (33%) of the respondents had changed drugs. The main reason given by those who had changed or stopped taking the drugs was side effects (28.8%). The other reasons are shown in Figure 1. There was a significant relationship between education levels and knowledge of the ARV drugs (*χ*^2^ = 13.067, df = 3, *p* = 0.004).

### Social support

The results indicate the immediate family was perceived most supportive, followed by the support group. The mean values from the various sources are immediate family, 3.7 ± 0.6; support group, 3.1 ± 0.8; extended family, 2.7 ± 1.1; friend, 2.7 ± 0.9; and community, 1.8 ± 1.0. Results on the type of support showed the following results: emotional support, 1.9 ± 0.3; material support, 1.8 ± 0.3; ARV support, 1.8 ± 0.4; and food support, 1.7 ± 0.3. Although the source of support was ranked quite high, the type of support was not highly ranked. About half of the respondents (51.8%) perceived the social support they received as good, whereas 48.2% perceived it as inadequate.

### Side effects

**FIGURE 2:**
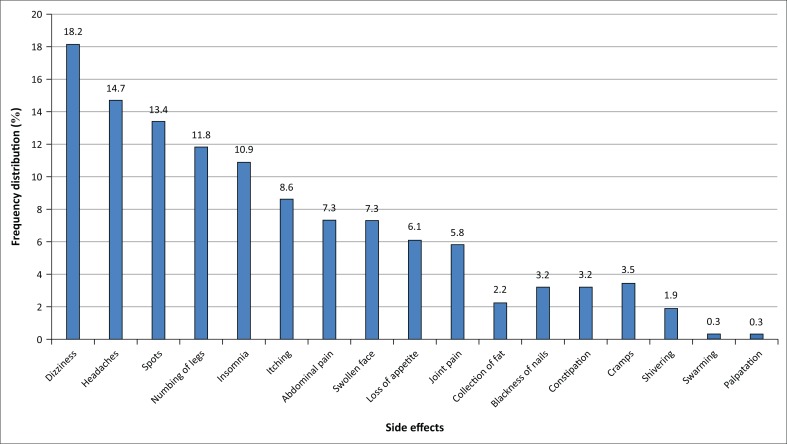
Frequency distribution (%) of the types of medication side effects of antiretroviral drugs reported by people living with HIV or AIDS in Machakos County, Kenya, 2015, *N* = 301.

All the respondents had experienced some side effects of the ARV treatment. About 22.4% experienced 1 side effect, 47.6% had 2–3 side effects and 30% experienced ≥ 4 side effects. The main side effects reported were dizziness 18.2%, headaches 14.7%, skin rashes 13.4%. The other side effects are shown in Figure 2.

### Factors associated with adherence

A Pearson’s chi-square test revealed that there was a significant relationship between marital status and the level of adherence (*χ*^2^ = 10.3, df = 3, *p* = 0.02). Divorced people tended to be more adherent. Out of the 27 divorcees, only 1 divorcee fell into the non-adherent category (< 95%). In contrast, there was no association between age, sex, education and adherence (*p* ≥ 0.05). There was a significant relationship between social support and adherence (*χ*^2^ = 8.960, df = 1, *p* = 0.003), with an odds ratio of 2.5 (CI 1.3–3.6), showing that patients with a good perception of social support were twice more likely to adhere to ARV drugs than those with a poor perception. There was a very significant relationship between side effects and adherence (*χ*^2^ = 68.312, df = 2, *p* ≤ 0.001). A binary logistic regression analysis was conducted to predict ARV adherence (adherent, non-adherent) using social support, side effects and marital status – variables that showed associations – as predictors. The statistical tests of each regression coefficients (i.e. bs) were tested using the Wald chi-square statistic. The intercept test result of *Y* = 0.825 is greater than *p* > 0.05, suggesting that an alternative model without an intercept needs to be applied. Goodness-of-fit test – Hosmer–Lemeshow (H–L) – was thus used. The Wald criterion demonstrated that marital status (*p* = 0.019) and burden of side effects (*p* ≤ 0.001) made a significant contribution to prediction of ARV adherence. Thus, the likelihood of a person adhering to medication was related to their marital status. Specifically, persons who are divorced were 11 times more likely to adhere to medication. Nagelkerke *R*^2^ of 0.406 shows that 41% of the variation in the ARV adherence was explained by these variables.

## Discussion

Most studies carried out on social support and side effects have been qualitative, descriptive or institution-based and have used self-reports. Our study was household-based which used pill count which is a more accurate method of assessing adherence. The purpose of the study was to establish the relationships between social demographic characteristics, social support, side effects and ARV adherence. Using the pill count, we were able to demonstrate relationships between marital status, social support and side effects (independent variables) and ARV adherence. Age, sex and education level were not independently associated with adherence. We showed that patients who perceived themselves to have more social support, patients who had fewer side effects and divorcees tended to be better adherents.

Using the respondents’ recall data, we found that adherence to ARV of 85.6% though slightly higher than those found in other African countries which ranged from 25% to 73.5%^6,7,8,9,10^ was below the standard of ≥ 95%. The adherence levels found in our study were within the range of 85% found in the United States.^5^ The adherence levels using the pill count of 58.6% in the household were lower than 67% using pill count at a hospital set-up.^11^ The fairly high levels found in our study could be explained by the fact that there are intensive HIV or AIDS interventions in this area. The findings that age, sex of the patient and education were not barriers to ARV adherence are consistent with those found in other studies from Kenya.^9,14^ Our study which found no significance of sex and age was inconsistent with others which showed that women were more adherent than men and younger age groups were more adherent than older ones.^13^ The probable reasons for the discrepancies in relating age to adherence could be because different authors used different cut-off points in categorising the age groups. Our study found that marital status was strongly associated with ARV adherence. The fact that being divorced was a key predictor from the logistic regression analysis shows it is an issue that requires more attention. We did not explore the reasons for this finding because the study was mostly quantitative. The answer to this requires qualitative studies. However, we speculate that this could be related to decision-making, where married women might be expected to ask for permission for care from their husbands whereas the divorced can make independent decisions. Although our study did not show an association between education level of the respondent and adherence, other studies have shown that low level of education and poor literacy affected the patient’s ability to adhere.^14,15^ Most patients were unaware of the name of the drugs they were using; there have been arguments that patients do not necessarily need to know the name of the drug as long as they could follow instructions.^28,29^

There are inadequate studies on the relationship between social support and adherence in Africa. Most of the studies on ARV and adherence are either descriptive or qualitative. These studies usually describe the type of support such as accompanying the patient to hospital or for appointments and reminding the patients to take their drugs and to get refills.^16,17,18^ Findings from other studies where family members have been found as the main sources of support are in conformity with our study.^17,19,20^ The role of support groups in our study was quite prominent showing their influence was greater than the extended family members. This indicates a move away from the traditional extended family or kinship who provided support, or where the family was perceived to be the most important contextual influence in the lives of PLWHAs. We established that inadequate social support was a barrier to ARV adherence. Quantitative studies done in Uganda, Tanzania and Botswana found no difference in adherence between those who had social support and those who did not have.^7,19^ Qualitative studies showed that social support was stated to be a factor in adherence.^20,22^ Our study further demonstrated that no type of support (material, financial, food and ARV support) was perceived to be of more importance than an other.

There are very few quantitative studies that show relationships between side effects and adherence. Qualitative studies have shown that side effects have consistently been associated with adherence.^16,21,22^ In our study, ‘burden of side effects’ or concentration of side effects contributed to ARV adherence. These findings, however, were not in conformity with a study conducted in South Africa, which showed no significant relationships between adherence and the concentration of symptoms, implying that despite the concentration of side effects patients were able to cope and continued being adherent.^23^

### Strengths and limitations

This study has several strengths. Most studies carried out on social support and side effects have been qualitative, descriptive or institution-based and have used self-reports. Our study was quantitative and household-based and used the pill count, a more accurate method of assessing adherence. A household-based study makes the findings more representative of the general community. We also used a theory-based approach – Health Belief Model – which we feel should be encouraged for both assessment and intervention. We found virtually no studies that have used the pill count at the household level. For a scientific study, the pill count proved more accurate for assessing the various relationships. Also, this study has demonstrated that the burden of side effects can influence ARV adherence. So far, it is the first study in Kenya to quantify ‘burden of side effects’ and its relationship to ARV adherence.

A major limitation of the study was that it did not include a comprehensive assessment of the multiple factors that hinder adherence. These include psychological and cognitive aspects. The study did not establish medication regimen factors that capture other dimensions of non-adherence to ARV such as schedule non-adherence which refers to not following a specific schedule for ARV medication (e.g. ‘3 times a day’ or ‘every 6 hours’). Instruction non-adherence which refers to not following special instructions for ARV medication, such as ‘take with food’ or ‘take on an empty stomach’ or dietary guideline adherence for patients reporting having special instructions for their ARV medications. Understanding medication is important because it motivates patients to overcome barriers of adherence. However, our study did not look at knowledge of ARV dosages, frequencies of the dosages and interval between those dosages. Our study did not include a qualitative research component that would have provided useful information on decision-making with regard to ARV adherence at the household level. These are some of the areas we recommend for further research.

## Conclusion

Levels of ARV drug adherence of ≥ 95% were not achieved, a pattern found in developed and developing countries. The social demographic characteristics of age, sex, education and social support did not contribute to ARV adherence. However, the likelihood of a person adhering to ARV medication was related to being divorced and ‘burden of side effects’ or the concentration of side effects.

## References

[1] Conway B The role of adherence to antiretroviral therapy in the management of HIV infection. J Acquir Immune Defic Syndr. 2007;45(Suppl 1):S14–S18. https://doi.org/10.1097/QAI.0b013e3180600766 17525686 10.1097/QAI.0b013e3180600766

[2] Gulick RM Antiretroviral treatment 2010: Progress and controversies. J Acquir Immune Defic Syndr. 2010;55(Suppl 1):S43–S48. 21045599 10.1097/QAI.0b013e3181f9c09e PMC3061404

[3] Beer L, Heffelfinger J, Frazier E, et al Use of and adherence to antiretroviral therapy in a large U.S. sample of HIV-infected adults in care, 2007–2008. Open AIDS J. 2012;6(Suppl 1: M21):213–223. 23056163 10.2174/1874613601206010213 PMC3465862

[4] Bangsberg DR Less than 95 percent adherence to nonnucleoside reverse-transcriptase inhibitor therapy can lead to viral suppression. Clin Infect Dis. 2006;43(7):939–941. https://doi.org/10.1086/507526 16941380 10.1086/507526

[5] Martin M, Del Cacho E, Codina C, et al Relationship between adherence level, type of the antiretroviral regimen, and plasma HIV type 1 RNA viral load: A prospective cohort study. AIDS Res Hum Retroviruses. 2008;24(10):1263–1268. https://doi.org/10.1089/aid.2008.0141 18834323 10.1089/aid.2008.0141

[6] Mitiku H, Abdosh T, Teklemariam Z Factors affecting adherence to antiretroviral treatment in Harari National Regional State, Eastern Ethiopia, ISRN AIDS, 2013;2013, Article ID 960954, 7 pages, 2013. https://doi.org/10.1155/2013/960954 10.1155/2013/960954 PMC3773384 24052892

[7] Hardon A, Davey S, Gerrits T, et al From access to adherence: The challenges of antiretroviral treatment [homepage on the Internet]. Geneva: WHO Press; [cited 2006 Oct 19]. Available from: http://www.who.int/medicines/publications/challenges_arvtreatment15Aug2006.pdf

[8] Potchoo Y, Tchamdja K, Balogou A, Pitche V, Guissou IP, Kassang EK Knowledge and adherence to antiretroviral therapy among adults people living with HIV/AIDS treated in the health care centers of the association ‘Espoir Vie Togo’ in Togo, West Africa. BMC Clin Pharmacol. 2010;10:11 https://doi.org/10.1186/1472-6904-10-11 20849595 10.1186/1472-6904-10-11 PMC2949664

[9] Talam NC, Gatongi P, Rotich J, Kimaiyo S Factors affecting antiretroviral drug adherence among HIV/AIDS adult patients attending HIV/AIDS clinic at Moi Teaching and Referral Hospital, Eldoret, Kenya. East Afr J Public Health. 2008;5(2):74–8. 19024414

[10] Uzochukwu B, Onwujekwe O, Onoka A, Okoli C, Uguru N, Chukwuogo O Determinants of non-adherence to subsidized anti-retroviral treatment in southeast Nigeria. Health Policy Plan. 2009;24:189–196. https://doi.org/10.1093/heapol/czp006 19276155 10.1093/heapol/czp006

[11] Maggiolo F, Ravasio L, Ripamonti D, et al Similar adherence rates favor different virologic outcomes for patients treated with nonnucleoside analogues or protease inhibitors. Clin Infect Dis. 2005;40:158–163. https://doi.org/10.1086/426595 15614706 10.1086/426595

[12] Reisner L, Matthew J, Skeer M, Perkovich B, Johnson CV, Safren SA A review of HIV antiretroviral adherence and intervention studies among HIV-infected youth. Top HIV Med J. 2009;17(1):14–25. PMC3752381 19270345

[13] Sasaki Y, Kakimoto K, Dube C, et al Adherence to antiretroviral therapy (ART) during the early months of treatment in rural Zambia: Influence of demographic characteristics and social surroundings of patients. Ann Clin Microbiol Antimicrob. 2012;11:34 https://doi.org/10.1186/1476 23270312 10.1186/1476-0711-11-34 PMC3599627

[14] Manyeki RW The influence of psychosocial factors on HIV antiretroviral drugs adherence among young people in Nakuru central district Kenya [unpublished dissertation]. 2012.

[15] Munthali CS Acceptability of antiretroviral drugs among adults living in Chawama, Lusaka [Thesis]. University of Zimbabwe; 2010.

[16] Nsimba SED, Irunde H, Comoro C Barriers to ARV adherence among HIV/AIDS positive persons taking anti-retroviral therapy in two Tanzanian regions 8–12 months after program initiation. J AIDS Clin Res. 2010;1:111 https://doi.org/10.4172/2155-6113.1000111

[17] Knodel J, Kespichayawattana J, Saengtienchai C, Wiwatwanich S The role of parents and family members in ART treatment adherence: Evidence from Thailand. Res Aging. 2010;32:19–39. https://doi.org/10.1177/0164027509348130 20221313 10.1177/0164027509348130 PMC2835367

[18] Zuurmond M Adherence to ARVS: Challenges and successes [homepage on the Internet]. 2008 Available from: http://catalogue.safaids.net/publications/adherence-arvs-challenges-and-successes

[19] Li L, Lee S, Wen Y, Lin C, Wan D, Jiraphongsa C Antiretroviral therapy adherence among patients living with HIV/AIDS in Thailand. Nurs Health Sci. 2010;12(2):212–220. https://doi.org/10.1111/j.1442-2018.2010.00521.x 20602694 10.1111/j.1442-2018.2010.00521.x PMC2947817

[20] Kumarasamy N, Safren SA, Raminani SR, et al Barriers and facilitators to antiretroviral medication adherence among patients with HIV in Chennai, India: A qualitative study. AIDS Patient Care STDS. 2005;19:526–537. https://doi.org/10.1089/apc.2005.19.526 16124847 10.1089/apc.2005.19.526

[21] Barnett W, Patten G, Kerschberger B, Conradie K, Garone DB, Van Cutsem G Perceived adherence barriers among patients failing second-line antiretroviral therapy in Khayelitsha, South Africa. S Afr J HIV Med. 2013;14(4):170–176. https://doi.org/10.7196/SAJHIVMED.981

[22] Sumbi VM Assessment of factors influencing adherence to antiretroviral therapy at Nyeri Provincial Hospital in Central Kenya [unpublished MSc thesis]. 2010.

[23] Bhengu BR, Ncama BP, McInerney PA, et al Symptoms experienced by HIV-infected individuals on antiretroviral therapy in Kwazulu-Natal, South Africa. Appl Nurs Res. 2011;24:1–9. https://doi.org/10.1016/j.apnr.2009.01.001 20974052 10.1016/j.apnr.2009.01.001

[24] Ministry of Health, Kenya Kenya HIV county profiles, National Aids and STI Control Programme Statistics [homepage on the Internet]. 2014 Available from: http://www.nascop.or.ke/

[25] Jung SH Stratified Fisher’s exact test and its sample size calculation. Biom J. 2014;56(1):129–140. https://doi.org/10.1002/bimj.201300048 24395208 10.1002/bimj.201300048 PMC3884832

[26] Stretcher V, Rosenstock IM The Health Belief Model In: Glanz K, Lewis FM, Rimer BK, eds Health Behaviour and Health Education: Theory, Research and Practice. San Francisco: Jossey-Bass; 1997.

[27] Duong M, Piroth L, Grappin M, et al Evaluation of the Patient Medication Adherence Questionnaire as a tool for self-reported adherence assessment in HIV-infected patients on antiretroviral regimens. HIV Clin Trials. 2001;2:128–135. https://doi.org/10.1310/M3JR-G390-LXCM-F62G 11590521 10.1310/M3JR-G390-LXCM-F62G

[28] Boateng D, Kwapong GD, Agyei-Baffour P Knowledge, perception about antiretroviral therapy (ART) and prevention of mother-to-child-transmission (PMTCT) and adherence to ART among HIV-positive women in the Ashanti region. BMC Women’s Health. 2013;13:2 https://doi.org/10.1186/1472-6874-13-2 23336813 10.1186/1472-6874-13-2 PMC3563602

[29] Duff P, Kipp W, Wild TC, Rubaale T, Okech-Ojony J Barriers to accessing highly active antiretroviral therapy by HIV-positive women attending antenatal clinic in a regional hospital in western Uganda. J AIDS. 2010;13:37 https://doi.org/10.1186/1758-2652-13-37 10.1186/1758-2652-13-37 PMC2954932 20863399

